# Trans-Fat Labeling in Packaged Foods Sold in Brazil Before and After Changes in Regulatory Criteria for Trans-Fat-Free Claims on Food Labels

**DOI:** 10.3389/fnut.2022.868341

**Published:** 2022-05-18

**Authors:** Beatriz Vasconcellos de Barros, Rossana Pacheco da Costa Proença, Nathalie Kliemann, Daniele Hilleshein, Amanda Alves de Souza, Francieli Cembranel, Greyce Luci Bernardo, Paula Lazzarin Uggioni, Ana Carolina Fernandes

**Affiliations:** ^1^Department of Nutrition, Health Sciences Center, Postgraduate Program in Nutrition, Federal University of Santa Catarina, Florianópolis, Brazil; ^2^Nutrition in Foodservice Research Center, Health Sciences Center, Federal University of Santa Catarina, Florianópolis, Brazil; ^3^Department of Nutrition, Health Sciences Center, Federal University of Santa Catarina, Florianópolis, Brazil; ^4^International Agency for Research on Cancer, World Health Organization, Lyon, France; ^5^Postgraduate Program in Collective Health, Department of Nutrition, Federal University of Santa Catarina, Florianópolis, Brazil

**Keywords:** trans-fat, partially hydrogenated oil, food ingredients, food labeling, nutrition labeling, food legislation, industrialized foods, census method

## Abstract

Consumption of industrially produced trans-fat acids (TFA) is a public health concern. Therefore, it is important that information on TFA in packaged foods be clearly informed to consumers. This study aimed to assess the evolution of TFA information presented in packaged foods sold in Brazil in 2010 and 2013, before and after the introduction of stricter regulatory requirements for TFA-free claims on food labels. A repeated cross-sectional study was performed through food label censuses of all packaged foods available for sale in two stores from the same supermarket chain, totaling 2,327 foods products in 2010 and 3,176 in 2013. TFA-free claims and information indicating TFA in the ingredients list and nutrition facts label were analyzed by descriptive statistics and Pearson’s chi-square test. There was a 14% decrease in the use of ingredients containing or potentially containing industrially produced TFA (i-TFA), according to analysis of the ingredients list. However, when analyzing foods by groups, it was found that this decrease was significant only for group A (bakery goods, bread, cereals, and related products; from 59 to 35%, *p* < 0.001). By contrast, food group F (gravies, sauces, ready-made seasonings, broths, soups, and ready-to-eat dishes) showed a 5% increase in i-TFA. The use of specific terms for i-TFA decreased between 2010 and 2013, but there was an increase in the use of alternative terms, such as vegetable fat and margarine, which do not allow consumers to reliably identify whether a food product is a possible source of i-TFA. There was an 18% decrease in the use of TFA-free claims in products containing or potentially containing i-TFA. However, almost one-third of foods sold in 2013 were false negatives, that is, foods reported to contain 0 g of TFA in the nutrition facts label or with TFA-free claims but displaying specific or alternative terms for i-TFA in the ingredients list. The results indicate that adoption of stricter requirements for TFA-free claims on food labels in Brazil helped reduce the prevalence of such claims but was not sufficient to decrease i-TFA in industrialized foods sold in supermarkets.

## Introduction

Trans-fat acids (TFA) are unsaturated fatty acids containing a double bond in the trans, rather than in the cis, configuration (1). These fatty acids can be formed via natural biohydrogenation processes in the gut of ruminant animals or through industrial processes, mainly hydrogenation (2). Moderate consumption of naturally occurring TFA may be considered safe and even healthy (3, 4), although findings are contradictory depending on the type of fatty acid (5, 6). On the other hand, the deleterious effects of consumption of industrially produced TFA (i-TFA) are well established. For this reason, the focus of this study is i-TFA. Partially hydrogenated oils (PHO), produced by hydrogenation of vegetable oils in the presence of a metal catalyst at high temperatures under vacuum, are the major sources of i-TFA (7). PHO are widely used by the food industry because of their high adaptability to different applications, as they possess neutral taste, are solid at room temperature and resistant to repeated frying (1, 8), and have low cost and high palatability (9).

According to Wanders et al. (10), Brazil has one of the highest consumptions of TFA worldwide. According to the 2008/2009 national survey, the average daily consumption of TFA is 2.4 g (11), although this intake may be even higher, considering that the survey is based on a two-day self-report. i-TFA are considered unsafe for human consumption in any quantity, given their association with several diseases, mainly those of cardiovascular nature, as well as with increased risk for all-cause mortality (12–15). Because of the negative impacts of i-TFA on human health, the World Health Organization (WHO) has issued recommendations against their consumption since 2004 (16) and declared their eradication a goal to be achieved by 2023 (17). With this aim in view, the WHO launched in 2018 the REPLACE action package, a guide detailing strategies to help countries eliminate i-TFA from the food production system (18). The guide provides recommendations for the creation and monitoring of public policies and inclusion of i-TFA in nutrition labeling. In Brazil, nutrition information on TFA has been mandatory since 2003 (19). Furthermore, the country has taken the actions recommended by WHO, having modified regulatory criteria for TFA-free claims on food labels (2012) and, more recently, mandating the reduction and elimination of i-TFA from the food system (17, 20). This latter regulation, passed in 2019, bans the use of PHO in industrial food processes and food services as of 2023 (20). However, while this regulation does not come into effect, food labels remain the primary means of informing consumers about TFA in packaged foods (21), which is an important factor influencing food decisions and choices (22).

According to Brazilian and Mercosur legislation, food labeling is mandatory for all ready-for-sale foods packaged in the absence of the consumer. Labels must contain descriptive information on packaged foods and beverages, including the ingredients list (23) and nutrition labeling (19). The nutrition facts label must contain quantitative descriptions of energy value, carbohydrates, proteins, total fat, saturated fat, TFA, and sodium (19). Although TFA information is mandatory, current legislation has limitations that make it difficult for consumers to correctly identify TFA in food products by using food labels (24). One such example is the possibility for manufacturers to declare a TFA content of 0 g in the nutrition facts label when the product contains less than or equal to 0.2 g of TFA per serving, without any distinction between naturally occurring TFA and i-TFA.

In Brazil, there are specific requirements regulating the use of nutrition claims, which are defined as any representation that implies that a food product has specific nutritional properties (19). The 2003 regulation on nutrition claims was updated in 2012, when new conditions for application of nutrition claims on food labels were enacted. Some changes were related to TFA claims: the 2012 regulation states that manufacturers can label a product with the terms “zero trans,” “0% trans-fat,” or “does not contain trans-fat” if the food product contains less than or equal to 0.1 g of TFA per serving (whereas the limit of the 2003 regulation was 0.2 g), be it naturally occurring or i-TFA, provided that the sum of TFA and saturated fat does not exceed 1.5 g per serving (25). The regulation passed in 2019 does not change the parameters for TFA-free claims. For this reason, manufacturers will still be able to use TFA-free claims even when foods are sources of this type of fat, until TFA is completely banned.

It can be seen that, according to Brazilian legislation, manufacturers can declare a TFA content of zero in the nutrition facts label (19) or use a TFA-free claim (25) even for food products containing i-TFA. The only way for consumers to ascertain whether a product contains i-TFA is by reading the ingredients list, which, as per Brazilian and Mercosur legislation (23), must describe, in descending order of proportion, all substances used in the manufacture or preparation of a food product. Furthermore, in all foods that contain fat, the list of ingredients must state whether it is vegetable or animal fat, without specifying the industrial process used, with the exception of butter (23). Identifying i-TFA by using the ingredients list, however, might not be an easy task, as shown by a study analyzing the label of 2,327 packaged foods sold in Brazil in 2010. A total of 23 different terms for ingredients possibly containing i-TFA were identified, 14 of which clearly referred to hydrogenation, whereas the remaining 9 generated uncertainty as to whether the food product contained i-TFA or not (26). Furthermore, according to Brazilian legislation (23), compound ingredients that correspond to less than 25% of the final product do not need to have their composition disclosed, which makes it even harder for consumers to have clear information about what they are buying.

It is important to monitor trends in TFA information presented on packaged food labels to assess the progression of this type of fat in foods available to the population (27). Despite the importance of this investigation, only three studies on the topic were identified, in which labels of different food groups were sampled in supermarkets and the data were used to determine the prevalence of TFA in packaged foods over time.

In Slovenia, Zupanic et al. (28) analyzed information on PHO in 8,557 prepackaged foods in 2015 and 14,072 prepackaged foods in 2017. The authors found that, despite the decrease of 2.4% in PHO information on food labels, a considerable proportion of foods still contained PHO in 2017, evidence that voluntary regulatory pressures might be insufficient to obtain satisfactory results concerning the use of PHO in packaged foods. In the Netherlands, Bend et al. (29) monitored, from 2006 to 2016, the nutrient content of 4,343 food products containing a logo used to identify healthier options. Overall TFA contents decreased by 48%, with a significant difference in 11 of the 27 food groups analyzed.

Lastly, a Canadian study conducted by Franco-Arellano et al. (30) analyzed the prevalence of PHO, hydrogenated fats, and/or both in 15,286 packaged food items in 2013 and 17,589 items in 2017 and determined the TFA content of the products. The use of TFA decreased significantly (from 0.8 to 0.2, 5 to 2.4, and 5.7 to 2.6%, respectively). As a result, there was a significant decrease in TFA content in almost all food groups, except for bakery products, for which there was an increase in TFA content. The authors concluded that voluntary measures to reduce TFA were not effective in Canada.

However, none of these studies investigated the effect of changes in regulations on TFA content, and there are no follow-up studies assessing TFA information on food labels in Brazil. In view of these observations, this study aimed to compare the prevalence of TFA on packaged food labels sold in Brazil in 2010 and 2013 and analyze how the change in legal requirements for TFA claims enforced in 2012 impacted TFA information on labels in packaged foods over time.

## Materials and Methods

A comparative analysis was performed using a repeated cross-sectional design, with two censuses of food labels, carried out in 2010 and 2013, in two stores from a large supermarket chain in Brazil.

### Location and Data Collection

The initial sample included all labels of all packaged foods available for sale in two large supermarkets belonging to one of the 10 largest chains in Brazil. Places of data collection were chosen intentionally, aiming for the inclusion of foods from different brands found throughout the country. Data collection was carried out in two stores in 2010 and 2013. Of all manufacturers included in the database, at least 69% in 2010 and 70% in 2013 supplied their food products nationwide.

All packaged food products available for sale that met the criteria established by the Brazilian and Mercosur regulations on food labeling (19) were included in the censuses of food labels. Products not included in the study were those covered by different regulations (e.g., food for babies and toddlers) or those that did not require mandatory nutrition labeling (e.g., bakery products produced, packaged, and labeled in-store and meat and cheese cuts packaged and labeled in-store). Fresh foods, such as meats, vegetables, and fruits, are not subject to Brazilian legislation on food labeling and were therefore excluded from the study. Products without added fat in their composition, such as flours and rice, were also excluded.

### Data Collection

**FIGURE 1:**
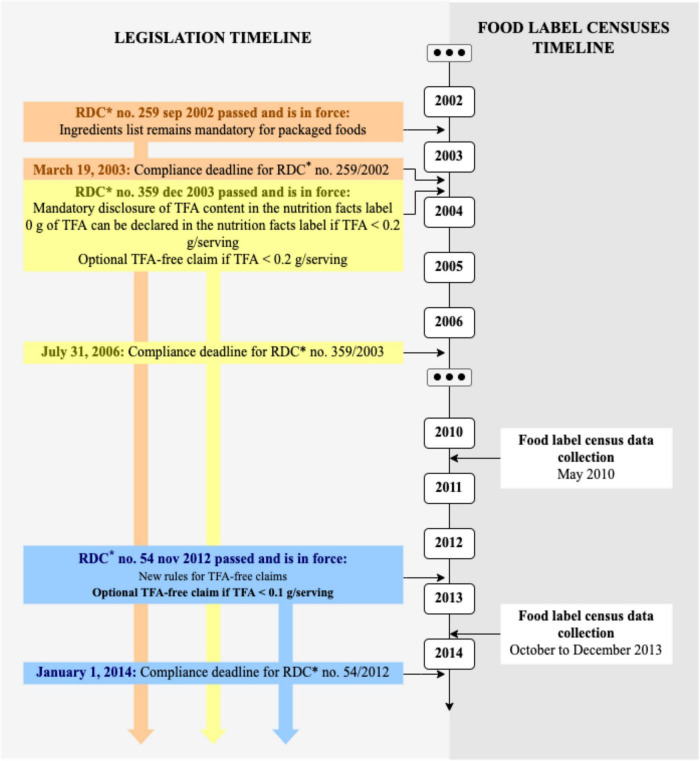
Timeline (2002–2014) of legislation on trans-fat acid (TFA) food labeling and food label censuses. *RDC, Resolution of the Collegiate Board of Directors of the Brazilian Health Regulatory Agency.

Data collection was authorized by supermarket managers, who signed an informed consent form, and carried out by nutritionists and undergraduate and graduate students in Nutrition of the university where the study was conducted. All data collectors received theoretical and practical training. Collections took place in 2010 and 2013. Figure 1 provides details about TFA legislation in Brazil and the data collection timeline.

The same type of information was obtained, but different collection instruments were used. The following information was collected: food type (product and flavor, trade name, and brand), nutrition facts label (serving size, serving size in household measure, and information on TFA provided by the manufacturer), TFA-free claims, and ingredients list.

In May 2010, data were collected on site and manually recorded on a form. Collected data were input into two separate databases, which were later checked for errors and validated. In 2013, data collection took place between October and December using the same form applied in 2010 but adapted to EpiCollect plus software, installed on Samsung Galaxy^®^ Note 8.0 tablets. All sides of all packaged foods were photographed. Each collector was responsible for specific areas of the supermarket. Data was automatically transferred via Wi-Fi to EpiCollect plus software and subsequently exported to Microsoft Excel^®^ version 2010. For quality control of data, 10% of food products were randomly selected for comparison between data and product photographs. A weighted kappa test was performed to verify data, with a result of 0.99, indicating reliability. After the test, any inconsistencies were rectified.

### Data Analysis

Data from the 2010 food label census were analyzed by Silveira and collaborators in 2011 (26). For comparison of TFA information collected in the two food label censuses, 2013 data were analyzed in the same manner as 2010 data.

Food labels were evaluated using three indicators: (1) i-TFA terms, identified by reading the ingredients list of each food in the database, (2) declaration of TFA in the nutrition facts label, and (3) existence of TFA-free claims.

Using the 2010 database, Silveira et al. (26) identified ingredients that indicated the presence of TFA (specific terms) and ingredients that could contain i-TFA (alternative terms, such as margarine).

**FIGURE 2:**
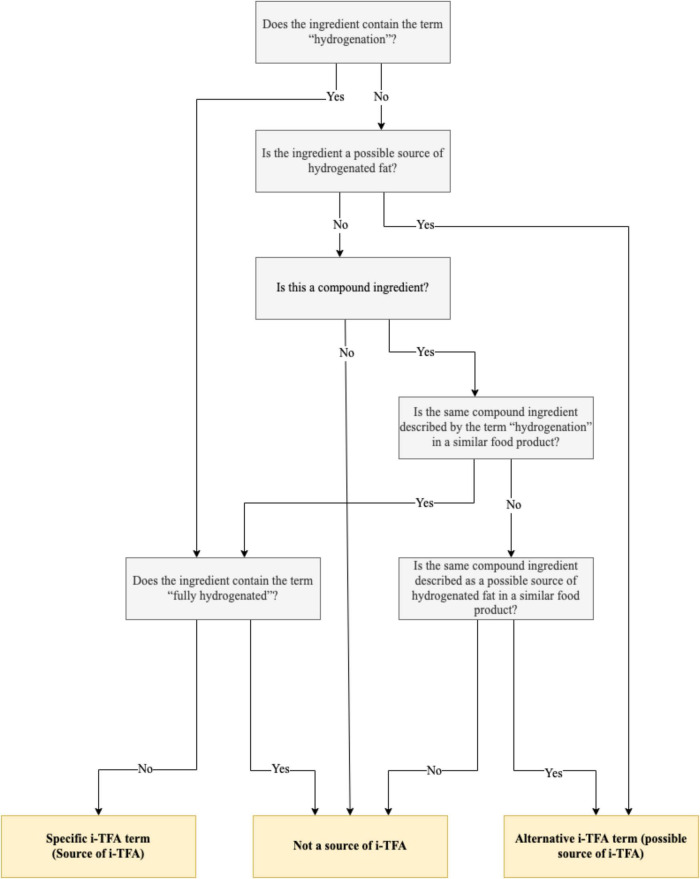
Decision criteria for determining whether a packaged food ingredient is a source of industrially produced trans-fat acids (i-TFA), a possible source of i-TFA, or not a source of i-TFA.

For the 2013 database, three observers separately analyzed the ingredients list of all food labels to identify specific and alternative terms for i-TFA listed in the 2010 food label census and other terms that had not been identified in 2010. All terms were reviewed by a fourth researcher, and conflicting classifications were discussed in a meeting with experts from the research group. For analysis of the ingredients list, it was considered that if the food product contained specific i-TFA terms, it would be counted as a food product containing i-TFA. If the same product contained another ingredient with alternative terms, it would not be counted again to avoid overlapping results. In the case of foods with compound ingredients of unknown composition, the product was considered to contain alternative terms when these compound ingredients were identified as a source or possible source of i-TFA in another similar food. For example, in some food products containing chocolate drops, the composition of the ingredient was specified in parentheses, allowing to identify chocolate drops as a source or possible source of i-TFA. Thus, all food products that contained chocolate drops but did not specify the composition of the ingredient were also considered to potentially contain i-TFA, as the ingredient was classified as an alternative term. Figure 2 illustrates the decision tree used for each ingredient.

Descriptive analysis of the 2013 database was performed concerning the existence of specific and alternative terms in the ingredients list; results are expressed as absolute and relative frequencies. We also present the absolute and relative frequencies of foods containing i-TFA information on the ingredients list, nutrition facts label, and TFA-free claims, stratified by food groups according to Brazilian and Mercosur regulations (19). Table 1 describes food groups with some examples.

**TABLE 1:** Description of packaged food labels sampled in 2010 and 2013, stratified by food groups according to the Brazilian and Mercosur regulation on nutrition labeling (36).

Group	Description	Examples of food items
A	Bakery goods, bread, cereals, and related products	Salty crackers, cakes without filling
B	Milk and dairy products	Dairy drinks, ice cream powder mix
C	Meats, eggs, and seafood products	Sausages, meat pastes, burgers, chicken nuggets
D	Oils, fats, and nuts	Mayonnaise, salad dressings
E	Sugars, sugary foods, and snacks	Sweet biscuits, cakes with filling, ice cream
F	Gravies, sauces, ready-made seasonings, broths, soups, and ready-to-eat dishes	Ready-to-cook and ready-to-eat dishes, sauce mix

A descriptive comparison of i-TFA terms identified in 2010 and 2013 food label censuses was performed. We also compared the prevalence of false negatives between i-TFA in the ingredients list and TFA information in the nutrition facts label and TFA-free claims between both food label censuses. A food product was considered a false negative if a source or possible source of i-TFA was identified in the ingredients list and the same product had a TFA-free claim or declared a TFA content of 0 g in the nutrition facts label.

Comparative analyses between food groups and the 2010 and 2013 food label censuses, as well as analysis of false negatives, were performed using Pearson’s chi-square test. All statistical analyses were conducted using Stata version 13.0 (StataCorp LP).

## Results

In the 2010 and 2013 food label censuses, 2,327 and 3,176 food labels were analyzed, respectively. There was an increase of 36% in food products that met the criteria for analysis of TFA information (potential sources of TFA) between 2010 and 2013. Meats, eggs, and seafood products (group C) was the group with the greatest increase in number of foods, mainly because of the increased variety of burger, chicken nugget, and sausage products.

In 2010, 1,318 citations of ingredients capable of containing i-TFA were identified, 25% of which were designated by specific terms and 75% by alternative terms. In 2013, 1,411 citations of ingredients likely to contain i-TFA were identified, 19% of which were specific and 81% of which were alternative.

As shown in Table 2, in 2010, 13 specific i-TFA terms were identified, which appeared a total of 335 times in ingredients lists. In 2013, 15 specific terms were identified, appearing 268 times. Comparatively, nine specific terms were introduced to food labels between 2010 and 2013. Seven terms that had been identified in 2010 were not found in food labels analyzed in 2013.

**TABLE 2:** Specific terms for sources of industrially produced trans-fat acids and their frequency of occurrence on the ingredients list of packaged foods sold in Brazilian supermarkets, as assessed by the food label census method in 2010 and 2013.

Specific terms	Census year	
	2010	2013
	*n* (%)	*n* (%)
Hydrogenated vegetable fat	305 (91.04)	228 (85.07)
Partially hydrogenated vegetable fat	1 (0.30)	8 (2.99)
Partially hydrogenated soybean and cotton oil	–	5 (1.87)
Hydrogenated palm oil	–	5 (1.87)
Vegetable hydrogenated fat	–	4 (1.49)
Partially hydrogenated soybean fat	2 (0.60)	3 (1.12)
Partially hydrogenated soybean oil	–	3 (1.12)
Hydrogenated fat	1 (0.30)	2 (0.75)
Hydrogenated soybean fat	4 (1.19)	2 (0.75)
Hydrogenated vegetable fat	–	2 (0.75)
Hydrogenated vegetable oil	8 (2.39)	2 (0.75)
Hydrogenated soybean oil	–	1 (0.37)
Hydrogenated vegetable oils	–	1 (0.37)
Partially hydrogenated soybean vegetable oil	–	1 (0.37)
Hydrogenated vegetable protein	–	1 (0.37)
Partially hydrogenated vegetable oil	6 (1.79)	–
Partially hydrogenated/interesterified fat	2 (0.60)	–
Liquid and hydrogenated vegetable oil	2 (0.60)	–
Hydrogenated	1 (0.30)	–
Hydrogenated vegetable margarine	1 (0.30)	–
Hydrogenated corn oil	1 (0.30)	–
Hydrogenated cotton, soybean, and palm oils	1 (0.30)	–
Total	335 (100.00)	268 (100.00)

In 2010, nine alternative terms were used 983 times in ingredients lists (Table 3). Of these, six were identified in 2013 in 909 occurrences. An additional 37 alternative terms were identified in 2013, with 234 occurrences. Therefore, the use of alternative terms increased in 2013 compared with 2010.

**TABLE 3:** Alternative terms for sources of industrially produced trans-fat acids and their frequency of occurrence on the ingredients list of packaged foods sold in Brazilian supermarkets, as assessed by the food label census method in 2010 and 2013.

Alternative terms	Census year	
	2010	2013
	*n* (%)	*n* (%)
Vegetable fat	771 (78.43)	728 (63.69)
Margarine	177 (18.01)	151 (13.21)
Condiment mix^a^	–	31 (2.71)
*Requeijão* ^b^	–	25 (2.19)
Chicken broth	–	24 (2.10)
Seasoning^a^	–	15 (1.31)
Dairy-based blend with vegetable fat	11 (1.12)	14 (1.22)
Vegetable fats	–	13 (1.14)
Vegetable margarine	9 (0.92)	13 (1.14)
Creamy *requeijão*^b^	–	11 (0.96)
Meat broth	–	10 (0.87)
White chocolate	–	10 (0.87)
Milk chocolate	–	9 (0.79)
Ready-made condiment^a^	–	9 (0.79)
Dairy-based blend	–	8 (0.70)
Chocolate chips	–	8 (0.70)
Dark chocolate	–	6 (0.52)
Chocolate	–	5 (0.44)
Dark chocolate-flavored stripes	–	5 (0.44)
Milk chocolate-flavored frosting	–	4 (0.35)
Milk chocolate chips	–	4 (0.35)
Chocolate-flavored chips	–	4 (0.35)
Triglyceride mixture	–	4 (0.35)
Chocolate-flavored biscuit	–	3 (0.26)
Chocolate syrup	–	3 (0.26)
Filling^a^	–	3 (0.26)
Dark chocolate-flavored frosting	–	2 (0.17)
Nature-identical ready-made condiment^a^	–	2 (0.17)
Chocolate-flavored sprinkles	–	2 (0.17)
Fat	1 (0.10)	2 (0.17)
Organic vegetable fat	–	2 (0.17)
Marshmallow	–	2 (0.17)
Hardened olive oil	–	1 (0.09)
Broiler broth	–	1 (0.09)
Chocolate sprinkles	–	1 (0.09)
Chocolate-flavored diet frosting	–	1 (0.09)
Chocolate-flavored confectionary sprinkles	–	1 (0.09)
Chocolate-flavored confectionary	–	1 (0.09)
Vegetable cream	5 (0.51)	1 (0.09)
Vegetable oils and fats	–	1 (0.09)
Cocoa chips	–	1 (0.09)
Complete seasoning powder	–	1 (0.09)
Seasoning similar to^a^	–	1 (0.09)
Sunflower vegetable fat	5 (0.51)	–
Soybean vegetable fat	1 (0.10)	–
Dairy beverage mix	3 (0.31)	–
Total	983 (100.00)	1,143 (100.00)

*^a^Different flavors (e.g., cheese, ham, sausage, barbecue) were grouped under the same category.*

*^b^Requeijão: Brazilian creamy cheese spread.*

As shown in Table 4, 51% (*n* = 1,175) of the 2,327 industrialized foods analyzed in 2010 were potential sources of i-TFA. This number decreased significantly in 2013, when 36% (*n* = 1,157) of the 3,176 food products were potential sources of this type of fat (*p* < 0.001).

**TABLE 4:** Prevalence of trans-fat acids (TFA) in packaged foods sold in 2010 and 2013 in Brazil, as determined by analyzing the ingredients list, nutrition facts label, and TFA-free claims on food labels.

Food group^a^	*N*		Ingredients list (total)		*p*	Ingredients list							Nutrition facts label						Nutrition claims		
						
						Specific terms			Alternative terms			*p*	>0 g TFA			0 gTFA					
	2010	2013	2010	2013		2010	2013	*p*	2010	2013	*p*		2010	2013	*p*	2010	2013	*p*	2010	2013	*p*
	*n*	*n*	*n* (%)	*n* (%)		*n* (%)	*n* (%)		*n* (%)	*n* (%)			*n* (%)	*n* (%)		*n* (%)	*n* (%)		*n* (%)	*n* (%)	
A	724	801	426 (59)	283 (35)	**<0.001**	105(14)	79 (10)	0.442	321 (44)	204 (25)	0.116	**<0.001**	138(19)	87 (11)	0.730	582 (80)	710 (89)	0.448	240 (33)	180 (22)	0.427
B	375	327	23 (6)	19 (6)	0.990	6 (2)	6 (2)	0.758	17 (4)	13 (4)	0.431	0.990	55 (15)	31 (9)	0.815	317 (84)	296 (90)	0.949	0 (0)	5 (1)	–*
C	97	461	43 (44)	32 (7)	0.370	3 (3)	5 (1)	0.857	40 (41)	27 (6)	–*	0.370	24 (25)	22 (5)	0.623	73 (75)	153 (33)	0.684	22 (23)	10 (2)	–*
D	77	141	31 (40)	24 (17)	0.175	17 (22)	14 (10)	0.632	14 (18)	10 (7)	**0.012**	0.175	13 (17)	1 (1)	0.650	64 (83)	137 (97)	0.355	24 (31)	41 (29)	0.112
E	753	1146	504 (67)	633 (55)	0.655	168 (22)	125 (11)	0.126	336 (44)	508 (44)	0.495	0.655	146 (19)	163 (14)	**<0.001**	606(80)	949 (83)	**<0.001**	199(26)	176 (15)	0.536
F	301	300	150 (50)	166 (55)	0.246	25 (8)	36 (12)	0.199	125 (41)	130 (43)	0.054	0.246	45 (15)	72 (24)	**0.010**	256 (85)	215 (72)	**0.015**	32 (11)	23 (8)	**<0.001**
Total	2,327	3,176	1,177 (51)	1,157 (36)	**<0.001**	324(14)	265 (8)	0.142	853 (37)	892 (28)	**0.006**	**<0.001**	421(18)	376 (12)	0.052	1,898 (82)	2,460 (77)	0.178	517 (22)	435 (14)	**<0.001**

*^a^Food groups were classified according to the Brazilian and Mercosur regulation on nutrition labeling (36): group A, bakery goods, bread, cereals, and related products; group B, milk and dairy products; group C, meats, eggs, and seafood products; group D, oils, fats, and nuts; group E, sugars, sugary foods, and snacks; group F, gravies, sauces, ready-made seasonings, broths, soups, and ready-to-eat dishes.*

*p-values were determined by Pearson’s chi-square test (95% confidence intervals).*

*Insufficient number of items in the category for analysis of statistical significance.

*Values in bold indicate that the differences are statistically significant at the 95% confidence intervals.*

There were no differences (*p* = 0.142) in the proportion of food products with specific i-TFA terms between food label censuses (14% in 2010 and 8% in 2013). However, there was a significant decrease, from 37 to 28%, in the proportion of food products containing alternative terms (*p* = 0.019). In considering only food products that contained i-TFA terms, it was found that the proportion of specific terms decreased from 28 to 23% and that of alternative terms increased from 72 to 77% between 2010 and 2013 (*p* < 0.001).

When considering the entire sample, we found a decrease in the proportion of foods containing i-TFA, but only because group A had a large and significant reduction. In analyzing food groups separately, we found a decrease in foods containing i-TFA only in bakery goods, bread, cereals, and related products (group A; *p* < 0.001), with no differences in the other groups.

From 2010 to 2013, there was an increase in the absolute number of food products without i-TFA in sugars, sugary foods, and snacks (group E) and bakery goods, bread, cereals, and related products (group A). In group E, there were no differences between foods that were considered potential sources of i-TFA. Thus, although there was an increase in the diversity of group E foods without i-TFA, the number of foods with i-TFA remained unchanged from 2010 to 2013.

From 2010 to 2013, there was no difference in the proportion of foods containing more than 0 g of TFA, as declared in the nutrition facts label, considering the entire sample (Table 4). In analyzing food groups separately, we found an increase from 80 to 83% (*p* < 0.001) in the proportion of foods with 0 g of TFA in group E, attributed to the increase in the diversity of foods without i-TFA (identified by analysis of the ingredients list). In the group gravies, sauces, ready-made seasonings, broths, soups, and ready-to-eat dishes (group F), there was an increase from 15 to 24% (*p* = 0.010) in food products that declared more than 0 g of TFA. It was observed that 1% of foods in 2010 did not report TFA information on the nutrition facts label, and no changes were observed between food label censuses in this regard.

The proportion of foods with TFA-free claims decreased from 2010 to 2013 (from 22 to 14%, *p* < 0.001). However, among food groups, such a decrease was significant only in the group gravies, sauces, ready-made seasonings, broths, soups, and ready-to-eat dishes (group F; from 10.6 to 7.7%, *p* < 0.001). Table 5 shows the prevalence of false negatives over time.

**TABLE 5:** Prevalence of false-negative information on trans-fat acids (TFA) in packaged foods sold in Brazil in 2010 and 2013, as identified by comparison of the nutrition facts label and TFA-free claims with the ingredients list.

Food group^a^	*N*		Nutrition facts label^b^		*p*	TFA-free claims^c^		*p*
					
	2010	2013	2010	2013		2010	2013	
	*n*	*n*	*n* (%)	*n* (%)		*n* (%)	*n* (%)	
A	724	801	299 (41)	196 (24)	0.188	128 (18)	54 (7)	0.495
B	375	327	18 (5)	19 (6)	0.885	0 (0)	0 (0)	–*
C	97	461	32 (33)	30 (6)	0.481	11 (11)	3 (1)	–*
D	77	141	25 (32)	23 (16)	0.281	0 (0)	3 (2)	–*
E	753	1146	365 (48)	481 (42)	**0.023**	141 (19)	100 (9)	0.843
F	301	300	124 (41)	96 (32)	0.181	20 (7)	16 (5)	**<0.001**
Total	2,327	3,176	863 (37)	845 (27)	**<0.001**	300(13)	176 (5)	**<0.001**

*^a^Food groups were classified according to the Brazilian and Mercosur regulation on nutrition labeling (36): group A, bakery goods, bread, cereals, and related products; group B, milk and dairy products; group C, meats, eggs, and seafood products; group D, oils, fats, and nuts; group e, sugars, sugary foods, and snacks; group F, gravies, sauces, ready-made seasonings, broths, soups, and ready-to-eat dishes.*

*^b^Prevalence of foods with specific or alternative terms for i-TFA in the ingredients list and 0 g of TFA declared in the nutrition facts label, treated as false-negative information.*

*^c^Prevalence of foods with specific or alternative terms for i-TFA in the ingredients list and TFA-free claims, treated as false-negative information.*

*p-values were determined by Pearson’s chi-square test (95% confidence intervals).*

*Insufficient number of items in the category for analysis of statistical significance.

*Values in bold indicate that the differences are statistically significant at the 95% confidence intervals.*

It was observed that the number of false negatives decreased (*p* < 0.001) from 2010 (37%) to 2013 (27%). However, such a reduction was significant only for foods of the group sugars, sugary foods, and snacks (group E; from 48% in 2010 to 42% in 2013, *p* = 0.023); no differences were observed for the other food groups. Furthermore, there was a reduction in the prevalence of false negatives between the ingredients list and TFA-free claims (13% in 2010 and 5% in 2013, *p* < 0.001). However, within groups, a significant reduction was observed only for gravies, sauces, ready-made seasonings, broths, soups, and ready-to-eat dishes (group F; 7% in 2010 and 5% in 2013, *p* < 0.001).

## Discussion

This study compared TFA information reported on the labels of packaged foods sold by a large supermarket chain in Brazil in 2010 and 2013 and analyzed how regulatory changes in 2012 (25) influenced the use of TFA in food formulations. In analyzing the ingredients list, we observed a decrease from 51 to 36% in the total number of foods containing i-TFA between 2010 and 2013. However, this result should be considered carefully, given that, within food groups, such a decrease in products with i-TFA was only significant for bakery goods, bread, cereals, and related products (group A). Of note, group A had the largest decrease in products with i-TFA, which might have influenced the results of the entire sample. Besides, there was an increase in the absolute number of foods containing i-TFA in groups E and F from 2010 to 2013. Thus, despite the decrease in the proportion of foods with i-TFA between food label censuses, it cannot be said that consumers had a higher possibility of choosing an i-TFA-free food, considering all available foods, in 2013 as compared with 2010.

In a survey conducted in the United States of America, Rahkovsky et al. (31) analyzed 37,628 labels from a database made available by the food industry for the 2005–2010 period. The authors observed that, despite the significant decrease in the amount of i-TFA, the reported i-TFA content was considered high over time, amounting to about 1.52 g per serving. In the present study, there was no decrease in the number of foods listing TFA in the nutrition facts label between 2010 and 2013. Therefore, according to information presented in the nutrition facts label, there was no improvement in the proportion of foods considered potential sources of TFA between food label censuses.

It was observed that the use of specific and alternative terms in all foods decreased from 14% in 2010 to 8% in 2013 and from 39% in 2010 to 30% in 2013, respectively. However, for foods that were sources of i-TFA, there was an increase in the proportion of alternative terms, from 78% in 2010 to 82% in 2013. In other words, the possibility that the information shown on the food label did not correctly inform about i-TFA content increased. According to Silveira et al. (26), the lack of standardization in i-TFA terms may confuse consumers about the real composition of food, possibly inducing consumers to inadvertently choose foods containing i-TFA.

A study conducted in 2017 in Brazil analyzed 11,434 food and beverage labels (32). One-fifth of the food products were found to be potential sources of i-TFA, and 4.1% of i-TFA terms were identified as specific. Such percentages were lower than those found in the present study: 14% in 2010 and 8% in 2013. The 2017 study found that 14.6% of labels declaring i-TFA contained alternative terms. However, the method of the referred study differed from that of the current one: only “margarine,” “vegetable fat,” and “vegetable cream” were considered as alternative terms in the 2017 study. Here, 9 alternative terms were identified in 2010 and 37 in 2013. Given that the 2017 study only considered three alternative terms, it can be inferred that there was an underestimation of i-TFA content reported on food labels.

Regarding false negatives, despite changes in the criteria for using TFA-free claims in 2012 (25), it is still possible to report 0 g of TFA on the nutrition facts label if the content of TFA is less than 0.2 g per serving (19), which might confuse consumers (26). Such a weakness in legislation was confirmed by Hissanaga-Himelstein et al. (33), who determined the composition of saturated fat and i-TFA in biscuits and breads sold in Brazil by gas chromatography and compared the results with information reported on food labels. It was revealed that 92% of the evaluated products contained i-TFA, although only 33% reported this information on the nutrition facts label.

In Shanghai, Kong et al. (34) investigated the nutrition information of packaged foods in 2007/2008 and 2012/2013. Of the 1,995 foods analyzed, 77% would be required to report the TFA content in the nutrition facts label because they contained PHO, but only 7% disclosed this information. In the study of Wang et al. (35), the labels of 895 margarines sold in the United States of America were investigated over time (2001, 2006, and 2011). In 2001, 2.3% of all margarine types included i-TFA-free claims on the label. After a technical regulation that made it mandatory to inform the TFA content in the nutrition facts label was passed in 2006, the number of claims rose to 6.5%. Presumably, after reformulation of food products resulting from changes to legislation, the number of claims decreased to 3.1% in 2011. These findings show that public policies that do not allow false notification of TFA-free claims in the nutrition facts label or front-of-pack labels may promote the reformulation of foods with low i-TFA contents.

In the present study, we identified a decrease in the number of false-negative foods (10% reduction in the nutrition facts label and 8% reduction in TFA-free claims). However, despite this decrease, about one-third of foods containing i-TFA in 2013 received this classification; therefore, these food labels did not provide adequate information that would allow consumers to choose between consuming i-TFA or not. There was a greater decrease in the number of false negatives between TFA-free claims and the ingredients list than between the nutrition facts label and the ingredients list. This result might be due to changes in criteria for the use of TFA-free claims on food labels, effective as of 2012 (25): the TFA content permitted for displaying TFA-free claims in 2012 is half of that allowed in 2010. The 2019 regulation banning i-TFA in Brazil does not change the parameters for using TFA claims, allowing manufacturers to use a TFA-free claim if the food product contains less than 0.1 g of (naturally or industrially produced) TFA per serving and the total amount of TFA and saturated fat does not exceed 1.5 g per serving. For this reason, false negatives will continue to exist in the Brazilian market until i-TFA is completely banned.

Another limitation of Brazilian legislation is that it allows serving sizes to differ in ± 30% from the reference value (36). Kliemann et al. (37) observed that serving size might be associated with the declaration of i-TFA in nutrition information in Brazilian food products. Half of the 2,020 foods analyzed were likely to contain i-TFA according to the ingredients list, but analysis of the nutrition facts label indicated that about 40% were false negatives. TFA content and number of false negatives increased with increasing serving sizes up to the maximum value allowed, decreasing for foods with serving sizes above the threshold. According to Machado et al. (38), the use of smaller serving sizes and fractionation of serving sizes in household measures are some of the strategies used to avoid informing TFA content in the nutrition facts label in Brazil.

For sugars, sugary foods, and snacks (group E), there was an increase in the number of products containing i-TFA in the ingredients list and declaring a TFA content greater than 0 g in the nutrition facts label. On the other hand, there was a decrease in the number of false negatives. Aued-Pimentel et al. (39) analyzed the lipid content of 600 foods sold in Brazil between 2005 and 2018; it was identified that the TFA content of sugars, sugary foods, and snacks (group E) increased over time and was considered high (21 g of TFA/100 g).

Other studies have monitored i-TFA information on the labels of foods that could be classified as group E. Hooker et al. (40) analyzed the labels of 2,701 cookies in the United States and 965 cookies in Canada between 2006 and 2012 and found a decrease in i-TFA content in both countries. Zupanic et al. (28) monitored the food labels of 22,629 products between 2015 and 2017 in Slovenia and observed a decrease in i-TFA content in all food groups except cakes, muffins, and cookies, in which i-TFA contents remained high.

Steen Stender (41) has been collecting TFA information from biscuits, cakes, and wafers in supermarkets in 28 European countries and the former Soviet Union since 2012 (41–44). Studies evaluating TFA labeling over time have indicated a reduction in TFA content, although high TFA levels are still found in several countries. Furthermore, voluntary regulatory measures did not provide effective results in any country. In one of the studies, the authors highlighted the limitation of having considered only “partially hydrogenated fat” or similar terms for assessing TFA in the ingredients list (41). Therefore, the number of sources of i-TFA in the referred study would probably be higher if ingredients lists were analyzed individually and alternative terms were identified.

In the group gravies, sauces, ready-made seasonings, broths, soups, and ready-to-eat dishes (group F), there was an increase in the number of sources of i-TFA and no differences in the absolute number of foods between 2010 and 2013. We observed an increase in the number of foods with TFA, as identified in the nutrition facts label, indicating that there was an increase in the use of i-TFA in this group. In line with this finding, a decrease in the number of foods with TFA-free claims was observed, also possibly related to changes in legislation.

A strong aspect of the current study, as well as previous studies from our research group (26, 33, 38, 45), is the use of a reproducible, reference method to identify TFA information on food labels. One limitation is the fact that data were gathered from one Brazilian supermarket chain only. However, care was taken to choose a supermarket chain that was among the 10 largest in the country. Thus, foods sold in this supermarket chain were likely to have high national representativeness, added to the fact that, of all manufacturers, at least 69% in 2010 and 70% in 2013 supplied their food products nationwide. Although the data are from 2010 and 2013, as previously mentioned, the regulation passed in 2019 did not change the parameters for TFA labeling. Thus, consumers cannot be certain whether a given food product is a source of i-TFA until this type of fat is completely banned in 2023. Even though regulations for TFA-free claims are being revised worldwide, there were no studies analyzing the meaning of such changes for consumers through analysis of food labeling over time, until now. Future studies should monitor TFA information on Brazilian food labels to assess the impact of the latest regulatory measures on food products.

## Conclusion

The change in criteria for using TFA-free claims imposed by Brazilian legislation in 2012 led to a decrease in the proportion of foods containing TFA and with such claims on the label. However, regulatory changes did not significantly influence the composition of packaged foods between 2010 and 2013. Although there was a decrease in the proportion of foods containing i-TFA, separate analysis of food groups revealed a significant decrease only for bakery goods, bread, cereals, and related products, which might have influenced the results for the entire sample.

The occurrence of false-negative products can be attributed to gaps in Brazilian legislation on i-TFA labeling. Therefore, it is important to review legislation to exclude the possibility of declaring “zero trans” (in claims and in the nutrition facts label) when products contain an ingredient that is a source of i-TFA and to standardize terms used to designate i-TFA. Even though it is expected that i-TFA will be eliminated in Brazil in 2023, consumers have the right to adequate information about food. The new regulation does not change parameters for TFA-free claims; therefore, false negatives may continue to exist until TFA is completely banned. Furthermore, food manufacturers are responsible for ensuring that TFA information is clear enough for consumers to understand whether the product is a source of i-TFA or not.

Knowledge about the consequences of indiscriminate use and consumption of TFA needs to be carefully considered when listing other fats as substitutes for i-TFA. For instance, more scientific evidence is needed on the health effects of interesterified fats, given that, in the past, TFA was considered safe and even beneficial (46).

Stimulated by the importance of food labeling as a tool to ensure consumers’ right to information and promote healthier food choices, and the lack of follow-up studies on TFA information on food labels over time, this study provides evidence of the reality of Brazil regarding TFA information on food labels between 2010 and 2013. It also contributes to highlighting the existing weaknesses in Brazilian legislation concerning the declaration of TFA on food labels and is a potential source of data for comparing the situation in the country before elimination of TFA from food products, scheduled for 2023 (20).

## Data Availability Statement

The datasets presented in this article are not readily available because the database used in the article belongs to the Federal University of Santa Catarina and can be made available by request only in case of official partnership with the University. Requests to access the datasets should be directed to RP, nuppre@contato.ufsc.br.

## Author Contributions

BB was responsible for processing, analyzing, and interpreting data and drafting the manuscript. DH and AS contributed to data processing and analysis. GB and PU contributed to data interpretation and manuscript revision. RP was responsible for the design of the original study and contributed to student orientation and revision of the final manuscript. NK contributed to the design of the original study and to student orientation. AF was responsible for research coordination, student orientation, and revision of the manuscript. All authors approved the final version of the manuscript.

## Conflict of Interest

The authors declare that the research was conducted in the absence of any commercial or financial relationships that could be construed as a potential conflict of interest.

## Publisher’s Note

All claims expressed in this article are solely those of the authors and do not necessarily represent those of their affiliated organizations, or those of the publisher, the editors and the reviewers. Any product that may be evaluated in this article, or claim that may be made by its manufacturer, is not guaranteed or endorsed by the publisher.
